# Comprehensive Review on the Droplet Dynamics of an Acoustically Levitated Colloidal Droplet

**DOI:** 10.1021/acs.langmuir.6c00527

**Published:** 2026-04-21

**Authors:** Ramprasath Selvaraju, Aadil Kureshee, Kumaran Kannaiyan

**Affiliations:** † Faculty of Mechanical Engineering, Technion-Israel Institute of Technology, Technion City, Haifa 3200003, Israel; ‡ Mechanical Engineering Robotics Program, 543072 Guangdong Technion-Israel Institute of Technology, 241 Daxue Road, Shantou, Guangdong 515063, China; § Guangdong Provincial Key Laboratory of Materials and Technologies for Energy Conversion, GTIIT, Shantou, Guangdong 515063, China

## Abstract

In the context of clean energy transition, colloidal fuel droplets have attracted significant attention, owing to their high energy density and better combustion performance. Yet, considerable uncertainty remains regarding the dynamics and evaporation of colloidal droplets under the influence of an acoustic field. Among other methods, acoustic levitation provides a contactless platform for investigating droplet dynamics in the absence of solid interfaces. This Review presents a complete exploration of droplet dynamics in acoustically levitated colloidal droplets, from fundamentals to advanced applications. First, the fundamentals of acoustic levitation, including ultrasonic standing-wave generation and acoustic radiation forces that enable the stable, contact-free levitation of droplets, are outlined. The influence of acoustic levitation on the adjacent flow and thermal fields is considered, with an emphasis on acoustic streaming and other transport phenomena that substantially thin boundary layers and promote heat and mass transfers along levitated droplets. Subsequently, the influence of the acoustic field on droplet oscillations, droplet breakup and coalescence, and droplet evaporation behavior is discussed in detail. Across these aspects, recent studies have shown that the dynamics of colloidal droplets differ from those of pure fluids. Finally, current limitations and future research directions that could yield further insights into droplet dynamics are highlighted, as colloidal droplets have wide-ranging interdisciplinary applications.

## Graphical abstract

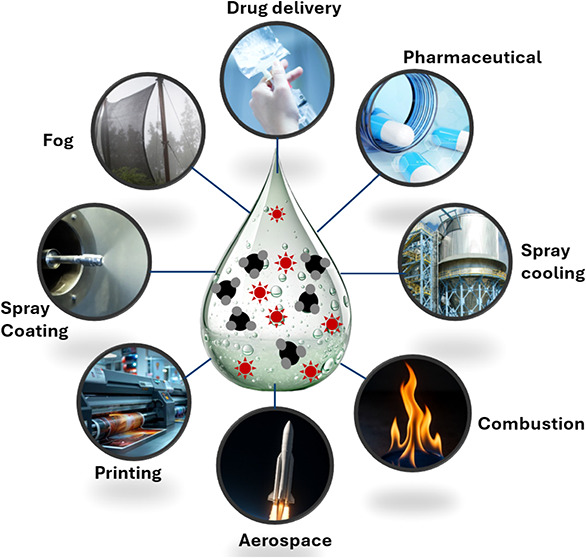

## Introduction

The Industrial Revolution and the exponential increase in fossil fuel consumption by modern civilization have dramatically increased anthropogenic greenhouse gas emissions, notably carbon dioxide, resulting in undesirable effects of climate change and global warming.^1^ In this context, liquid fuels have played a dominant role in land-based, airborne, and marine power generation, despite a gradual increase in the contribution of clean fuels.^2,3^ The key characteristic of liquid fuels is that they must undergo atomization, i.e., the disintegration of the bulk liquid into smaller droplets, evaporate, mix with the oxidizer, and then undergo combustion, resulting in the conversion of chemical energy into thermal energy. It is well-established that the initial steps in converting bulk liquid into fine droplets and the associated droplet dynamics play a crucial role in liquid-fuel-based power generation. Furthermore, liquid fuels are preferred in certain industries, such as aviation, marine, and heavy-duty power generation systems, owing to their high energy density and the difficulty of large-scale, rapid electrification. Consequently, there has been considerable interest in improving the understanding of droplet dynamics and gaining additional insights into their influence on subsequent processes.^4,5^ To achieve this goal, several methodologies have been adopted to study droplet dynamics through droplet suspension on a physical surface,^6–8^ free-falling droplet,^9^ or levitated droplet^10,11^ and decouple it from the external flow and thermal fields typically encountered in practical scenarios. Among these droplet study methods, acoustic levitation has regained considerable attention as other methods have their own limitations.^12–14^

On the other hand, with advancements in manufacturing micro- and nanoscale materials, there has recently been significant interest in micro- and nanoscale additives for liquid fuels to enhance heat release and combustion efficiency and, in turn, mitigate emissions.^4,15,16^ It is worth noting that most studies on droplet evaporation have focused on pure or emulsion fluids, and the mechanisms of their evaporation are reasonably well-established using various methodologies. However, the dispersion of discrete particles (micrometer- and nanometer-sized) alters the evaporation mechanism, which remains poorly understood. Thus, this study focuses solely on the evaporation mechanism of colloidal droplets investigated by using acoustic levitation. Accordingly, it differs from earlier reviews of acoustically levitated droplets. Nonetheless, colloidal droplet evaporation studies under the influence of an acoustic field (i.e., using the acoustic levitation method) for energy applications remain limited. Therefore, where appropriate, other forms of droplets (pure or emulsions) are used to discuss the underlying physical phenomena. This study aims to comprehensively review and provide insights into the dynamics of colloidal droplets, emphasizing fundamental concepts, limitations, and recent advances. Furthermore, it is also worth pointing out here that although the importance of droplet dynamics is highlighted above in the context of energy transition, colloidal droplet dynamics plays a crucial role in a wide range of scientific fields spanning from pharmaceuticals, printing technology, spray cooling/drying, carbon capture, healthcare drug delivery, etc., underscoring its importance across disciplines, as shown in Figure 1.

**Figure 1:**
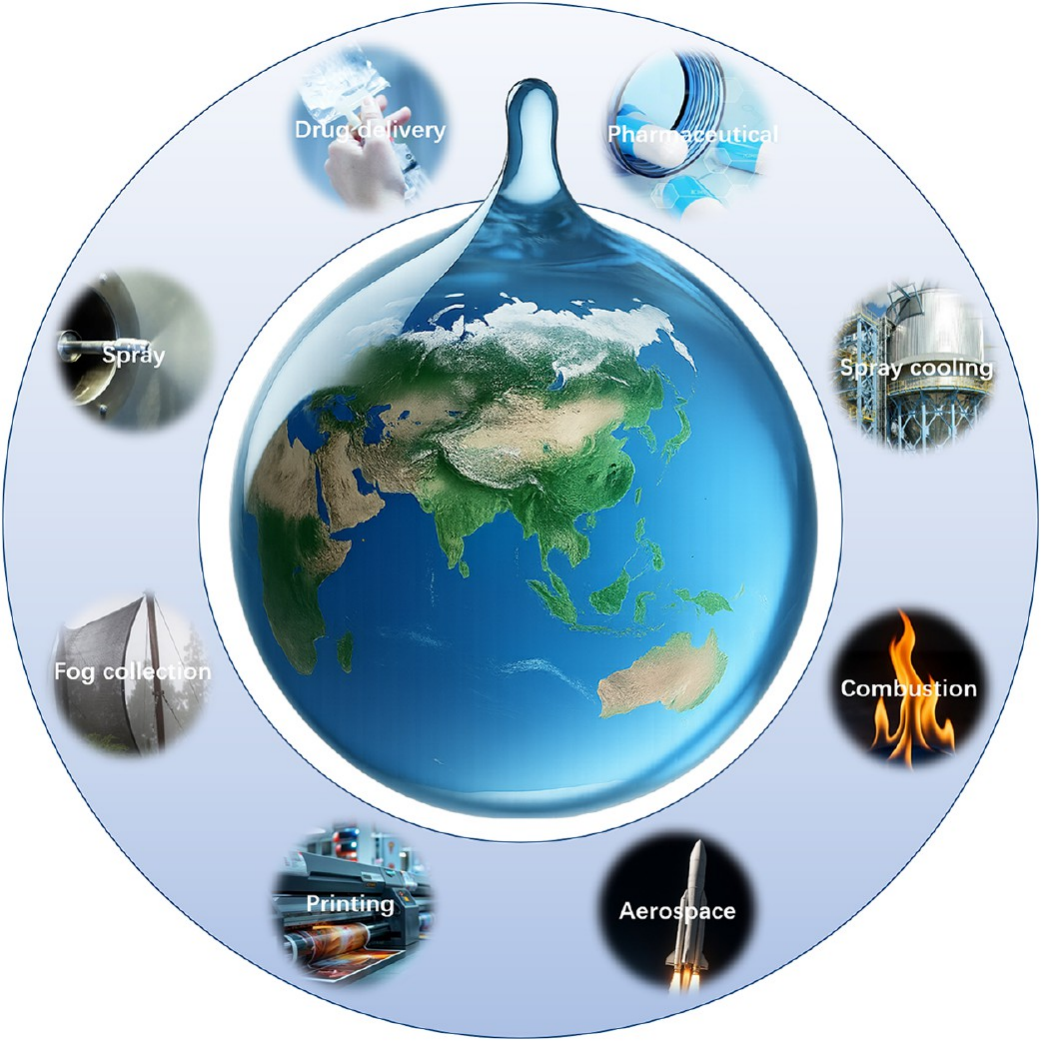
Application of colloidal droplet dynamics in interdisciplinary applications.

This comprehensive review begins with a brief overview of the fundamentals of acoustic levitation and its historical development, followed by an examination of its influence on the surrounding flow and thermal fields as well as on mass exchange. Next, the implications of these transport phenomena for individual droplet dynamics, breakup, and coalescence are discussed in detail. Finally, existing limitations and the path forward are highlighted, followed by a discussion of the potential future research directions.

### Acoustic Levitation (*AL*)

Acoustic levitation is a technique that uses the acoustic radiation forces of standing ultrasonic waves to suspend particles or droplets in air or other media. *AL* has emerged as a crucial nonintrusive tool in understanding the influence of droplet dynamics on the surrounding fluid, thermal and mass exchange, combustion, and material science. More importantly, the *AL* method, illustrated in Figure 2, is preferred due to the absence of physical boundary influence,^17,18^ which avoids contamination and interaction effects inherent in conventional suspension techniques. Therefore, *AL* is ideally suited for examining isolated droplets under rigorously controlled conditions.^19,20^ Furthermore, this method helps to decouple fluid dynamics and thermal and mass exchange from the combustion process. Acoustic levitation involves a complex interplay among fluid dynamics, acoustic waves, and thermodynamics that enables the suspension of a fluid droplet without direct contact. *AL* is typically achieved using ultrasonic waves emitted by a transducer, which are reflected from a single (or series of) concave reflector surface, forming a standing-wave field. These standing waves consist of alternating pressure nodes and antinodes, with a pressure node corresponding to a displacement antinode and vice versa. The time-averaged acoustic radiation force determines the stability of droplet levitation. This force can be better represented by the negative gradient of the effective acoustic potential, which can be better described by Gor’kov potential.^21^ For the minimum Gor’kov potential, the net radiation force vanishes, and a small displacement results in a restoring force that brings the droplet to a stable position. As a result, droplet stability is determined by the time-averaged acoustic radiation force. The acoustic levitation can also control the droplet rotation and speed for precise particle motion by varying the transducer array’s input voltage.^22^ Thus, in *AL*, the manipulation of acoustic pressure waves enables contactless positioning (i.e., suspension) of droplets at the pressure nodes.

**Figure 2:**
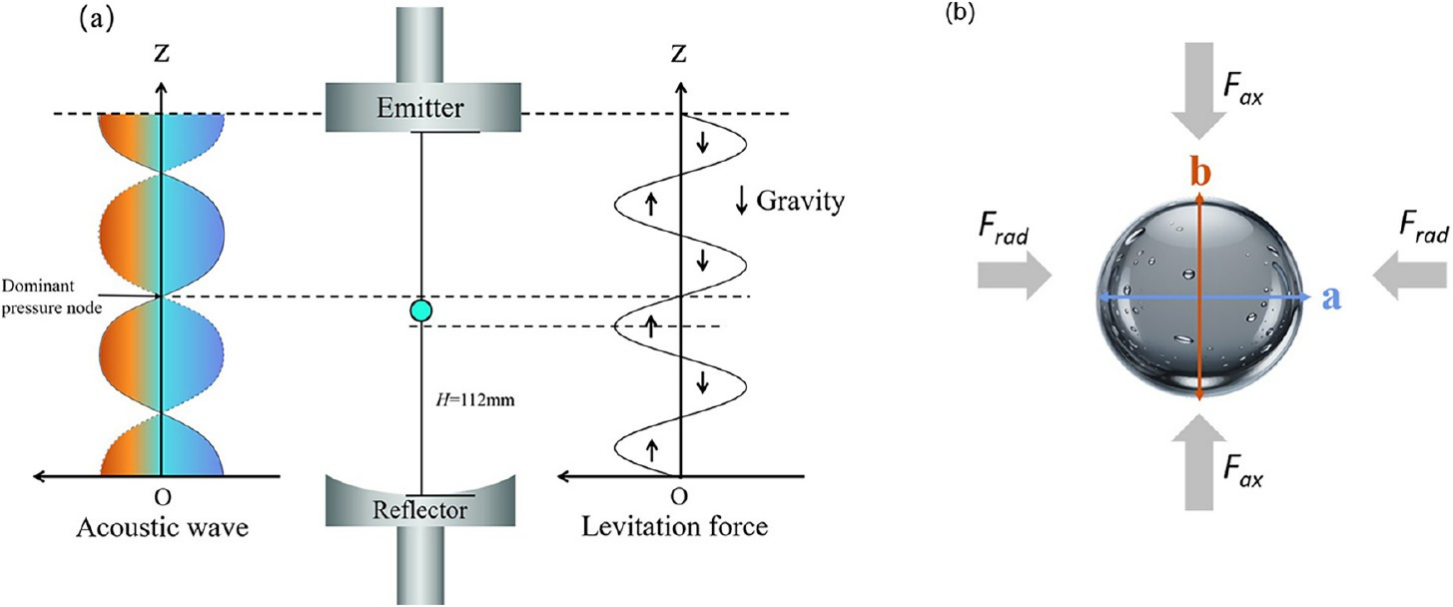
Schematic illustration of a single-axis levitator. (a) Schematic view of an acoustically levitated droplet and (b) forces acting on the levitated droplet with the aspect ratio parameters *a* and *b*.

### Acoustic Levitation: Balance of Forces

As depicted in Figure 2, droplets can be suspended at pressure nodes of standing acoustic waves through the balance of forces, *F* = −∇*U*. The acoustic radiation force (*F*) drives droplets toward regions of minimal potential energy (*U*), i.e., pressure nodes, enabling stable levitation.^21,23–25^ Here, *U* can be expressed as

$$U=\frac{4\pi a^{3}}{3}\left[f_{1}\left(\frac{\rho_{g}^{2}}{2\rho_{g}c_{g}^{2}}\right)-f_{2}\frac{3\rho_{g}<\nu >}{4}\ln\left(\frac{q_{0}}{q_{e}V_{a}}\right)\right],f_{1}=1-\frac{\rho_{g}^{2}c_{g}}{\rho_{d}c_{d}^{2}},\mathrm{and}\;f_{2}=\frac{2\left(\rho_{d}-\rho_{g}\right)}{\rho_{d}+\rho_{g}}$$

The above equation accounts for the balance of forces on the droplet surface, where *q*_0_ is the acoustic wavenumber in the gas phase (determined as *q*_0_ = ω/*c*_*g*_, where ω represents the angular frequency and *c*_*g*_ is the speed of sound), *q*_*e*_ *V*_*a*_ is a composite term representing (i) reference wavenumber (*q*_*e*_), often equivalent to *q*_0_ in simplified models and (ii) acoustic velocity amplitude (*V*_*a*_) typically denoted as *u*_0_ proportional to pressure amplitude (*p*_0_) via *u*_0_ = *p*_0_/(ρ_*g*_·*c*_*g*_), droplet radius (*a*), droplet physical properties (ρ_*d*_, *c*_*d*_), and ambient gas properties (ρ_*g*_, *c*, ν). The acoustic radiation force (*F*_rad_) is the primary force exerted by an acoustic wave on the suspended medium (droplet) in air. It is critical for maintaining (i) droplets in a stable levitated state,^23,26^ which, in turn, scales with the initial surface pressure (*p*_*i*_) and (ii) droplet sphericity. *F*_rad_ can be expressed through a modified Gor’kov potential equation^21^ using (eq 1)­

1 $$\begin{array}{l} F_{\mathrm{rad}}=\frac{\pi p_{i}^{2}V_{d}}{2\lambda \rho_{g}c_{g}^{2}}\left[5\Phi_{1}-2\Phi_{2}\right].\sin\left(2\mathrm{ky}\right)\\ \mathrm{where},\Phi_{1}=1-\frac{\rho_{g}c_{g}^{2}}{\rho_{d}c_{d}^{2}}\;\mathrm{and}\;\Phi_{2}=\frac{\left(\rho_{d}-\rho_{g}\right)}{2\rho_{d}+\rho_{g}} \end{array}$$

*F*_rad_ depends on the compressibility contrast (Φ_1_) and density contrast (Φ_2_), representing the difference in compressibility (here, “c” represents the speed of sound) and density, respectively, between the droplet fluid (subscript, *d*) and surrounding gas (subscript, *g*),^27,28^ acoustic wavelength (λ), and droplet volume (*V*_*d*_). These factors account for differences in droplet fluid properties (e.g., hydrocarbon and biofuels) and, in turn, their distinct behavior under the same acoustic field.^29,30^

### Acoustic Levitation: Droplet Characteristics

The spatial variation of *F*_rad_ induces droplet deformation as shown in Figure 2(b) and, in turn, the internal streaming (i.e., flow inside the droplet), which can directly influence the thermal (heat transfer) and mass (evaporation) exchange with the surrounding medium.^25,28^ Thus, a better understanding of the correlation is essential for elucidating the droplet evaporation mechanism in the *AL* method.^31^ The droplet deformation characteristics are quantified using the aspect ratio (*AR*) relationship, *AR* = 1.43 + 0.263*A*_0_ *c* – 0.1809*H* – 0.1332*A*_0_ *cH*, demonstrating the nonlinear coupling between incident acoustic-wave amplitude (*A*_0_) and the distance between transducer and reflector surfaces (*H*).^30,32^ By varying the *AL* parameters, it is possible to change the droplet aspect ratio (Figure 3) at the same pressure node.^33^ In Figure 3, the contour demonstrates the influence of ultrasonic amplitude and transmitter displacements in wavelength (λ) fractions on the resulting water droplet (*V*_*d*_ = 3.5 μL) aspect ratio modulated.

**Figure 3:**
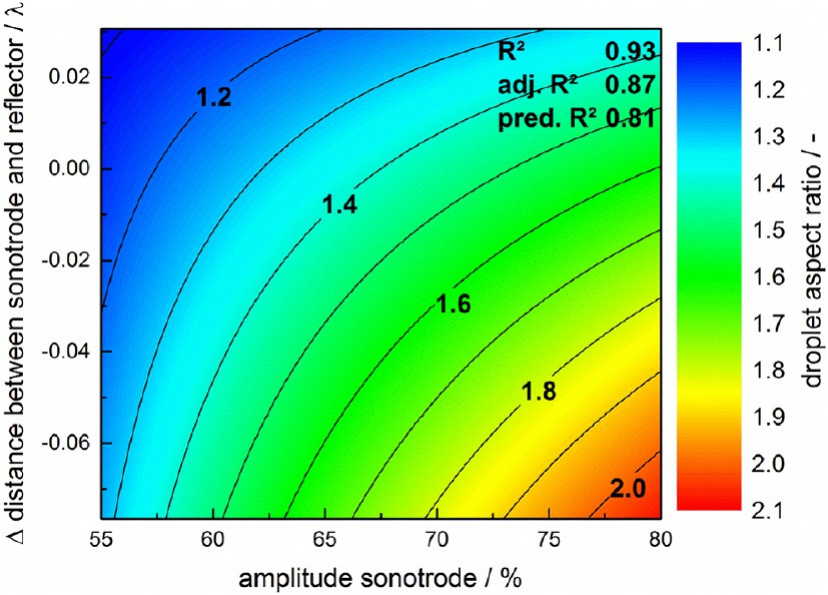
Influence of acoustic levitator amplitude and droplet characteristics on the droplet aspect ratio. Reproduced with permission from ref [Bibr ref33], 2025, Elsevier.

An oblate-spheroidal droplet shape is adopted in *AL* due to the inhomogeneous acoustic pressure field. Axial positioning force (*F*_ax_) dominates the radial force (*F*_rad_),^33^ compressing the droplet along the levitation axis and elongating it radially. This distortion is measured by the aspect ratio, which is an equilibrium between acoustic radiation pressure and droplet surface tension, distinguished by the acoustic Bond number (*Bo*_*a*_). The aspect ratio varies significantly with the acoustic field parameters. Furthermore, adjusting power, and therefore the sonotrode (i.e., transmitter) amplitude, can be helpful when acoustic-wave resonance conditions are not optimally met. This coupling makes it clear that several conditions govern evaporation, even if they are not explicitly stated. For instance, the aspect ratio of water droplets improves to 2.1 by reducing the distance between the transmitter and reflector to −0.077/λ, compared to standard standing-wave conditions while increasing the amplitude to 80%, as shown in Figure 3. Nonetheless, the minimum threshold of acoustic forces necessary to counteract gravitational forces is attained in this domain, and the experimental viability is not assured for all combinations.^33^ For example, in evaporating dodecane droplets, the aspect ratio exhibits a strong correlation with the initial droplet size, with small droplets (*d*_0_ < 450 μm) maintaining AR > 1 (ellipsoidal shape) with low-frequency oscillations (<25 Hz) throughout the evaporation process (Figure 4). In this figure, the aspect ratio oscillations in small (*d*_0_ < 450 μm) and large droplets (*d*_0_ > 450 μm) are shown. Zone I represents a stable regime, and Zone II represents an abrupt flattening before breakup. In contrast, larger droplets (*d*_0_ > 450 μm) display a near-constant AR in Zone I, followed by rapid amplification to a critical value in Zone II (∼50 ms), culminating in a catastrophic breakup. Higher heating rates shorten Zone I but do not affect the duration of Zone II. Nanoscale additives (0.1–0.5 wt % ceria) have negligible influence on these trends, underscoring the *AR* role as a precursor to breakup.

**Figure 4:**
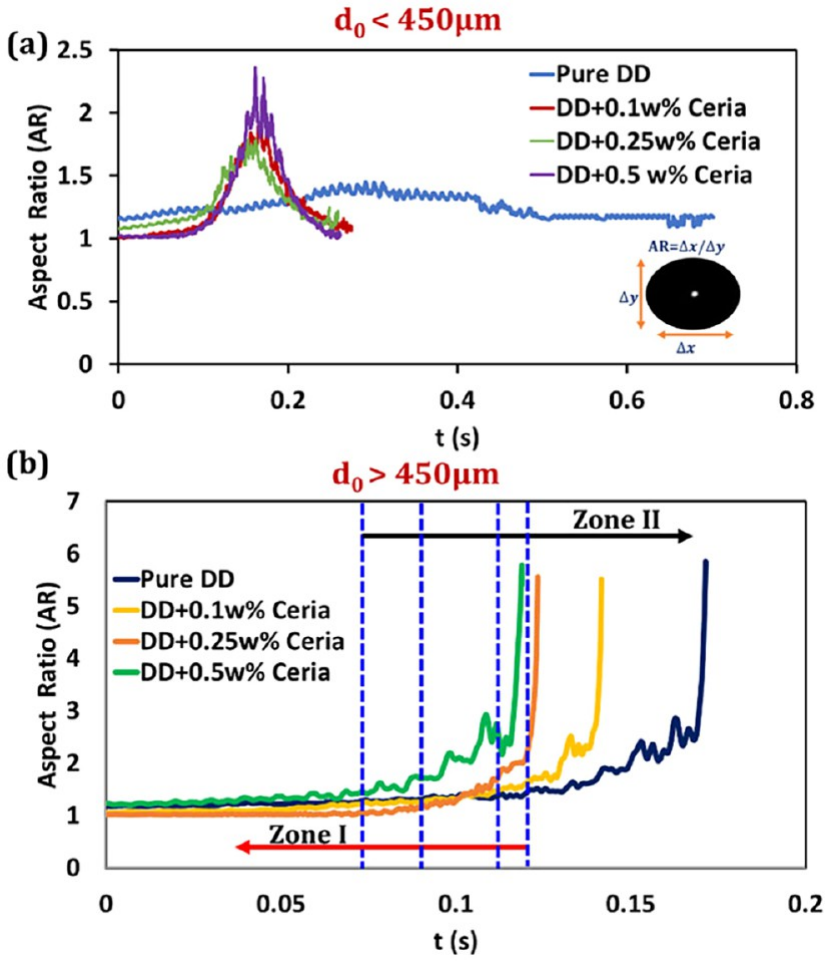
Influence of initial droplet diameter on the droplet aspect ratio of pure and colloidal droplets. (a) For initial droplet diameter (*d*_0_) < 450 μm and (b) *d*_0_ > 450 μm. DD: Dodecane. Reproduced with permission from ref [Bibr ref34], 2025, AIP Publishing.

As shown in Figure 5, it is essential to recognize that the conventional limit on the droplet size that can be acoustically levitated is smaller than half the acoustic wavelength (λ/2). This figure compares the acoustic potential and levitated droplet scales under normal and reduced gravity, along with the normalized sound pressure distribution for a solid sphere of λ/4 and λ/2. Reduced-gravity experiments enabled levitation, transport, and coalescence of droplets exceeding the half-wavelength limit, which were previously unstable under normal gravity.^35^ Nonetheless, the recent advances in transducer array technology^25,26,36^ have expanded the operational envelope under normal gravity, allowing stable levitation of droplet size up to a maximum of 0.6 λ through phase-modulation techniques.^21^ Transducer array technology refers to an arrangement of multiple sound-producing elements whose phase and amplitude can be controlled independently. A sample array system validating the acoustic potential and retention mechanism for trapping droplets is shown in Figure 6.^37^ In this figure, the acoustic field under focused ultrasound, a levitated droplet in a localized standing wave, and experimental and estimated values of sound pressure level comparison are shown. The acoustic field can then be tailored using multiple traps, steering, and phase-modulation techniques to levitate multiple or larger objects, significantly widening the operational limits of acoustic levitation.^37^ To better understand the droplet dynamics under the influence of acoustic waves, two factors need to be considered: (i) the coupling between the acoustic field and droplet dynamics and (ii) droplet oscillation under the influence of the acoustic wave.

**Figure 5:**
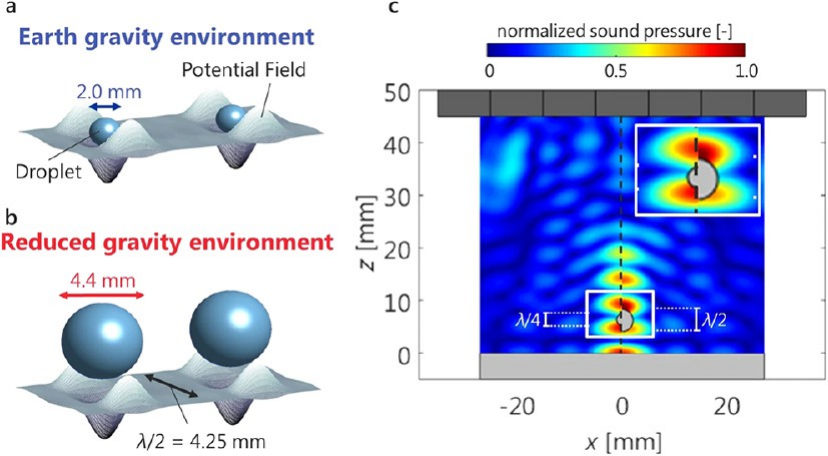
Influence of gravity on droplet levitation: (a) under normal gravity environment, (b) under reduced-gravity environment, and (c) normalized sound pressure distribution around a solid sphere. Reproduced from ref 36. Available under a CC-BY 4.0 license. Copyright 2019, Koji Hasegawa, Ayumu Watanabe, and Yutaka Abe.

**Figure 6:**
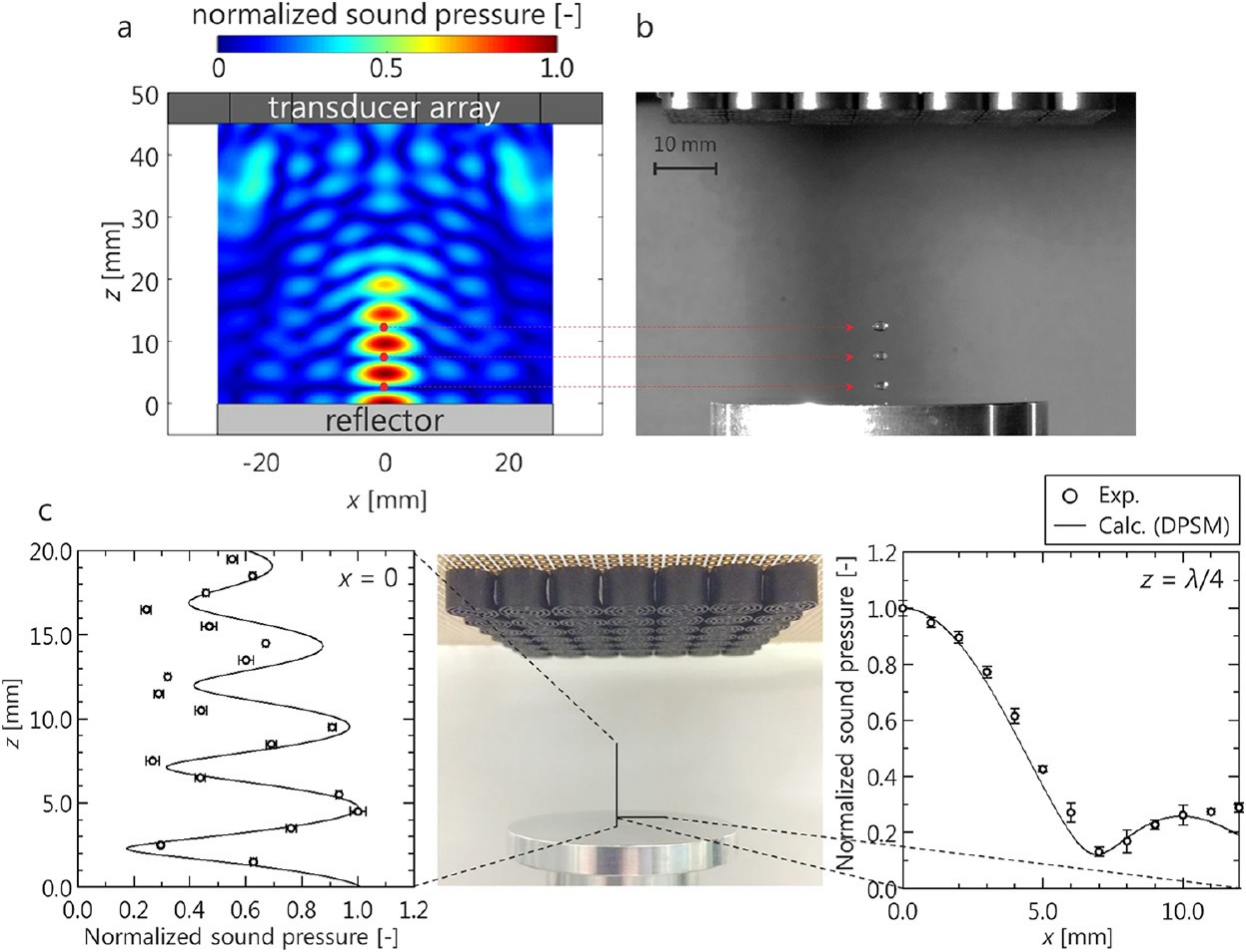
A sample array system demonstrating the acoustic potential and droplet retention mechanism (a) under focused ultrasound, (b) under localized standing wave, and (c) comparison of sound pressure levels between experimental and estimated values. Reproduced from ref 35. Available under a CC-BY 4.0 license. Copyright 2018, Ayumu Watanabe, Koji Hasegawa, and Yutaka Abe.

### Acoustic Field-Droplet Coupling

The coupling between droplet dynamics and the acoustic field is central for understanding and optimizing the *AL* parameters, as it governs both droplet stability and transport phenomena. The coupling produces a nonlinear feedback loop, in which the acoustic radiation force dictates the droplet’s position and shape, while the droplet characteristics, such as the size, composition, and oscillation, alter the ambient acoustic field. For instance, in acoustically levitated water droplets (2–4 mm diameter), the acoustic-wave-droplet coupling produces characteristic shape oscillations at the same frequency as that of the acoustic driving frequency (generally between 20 and 40 kHz), with deformation amplitudes being proportional to the acoustic energy density.^38^

The coupling process is highly dependent on the ratio of initial droplet diameter to acoustic wavelength (*D*_0_/λ), with maximum energy transfer at *D*_0_ ≈ λ/2.^39^ This relation indicates that stable levitation of smaller droplets requires a higher acoustic frequency.^19,26,32,37^ Therefore, for the stable levitation of a droplet, there is a size limit of λ/2, which is described by the potential well (distance between two pressure antinodes) for the single-axis acoustic levitator.^40^ The acoustic-droplet coupling also facilitates thermal and mass exchange significantly when compared to static (nonacoustic) conditions. These enhancements are primarily achieved through two mechanisms: (i) acoustic streaming that reduces the boundary-layer thickness approximately to 50 μm^33,41,42^ and (ii) internal thermoacoustic flows with velocities up to 10 mm/s augmenting the mixing process.^43^ In combustion applications, acoustic-wave-droplet coupling can be beneficial in shortening fuel ignition delays by 15–25% through enhanced fuel–air mixing.^44^ Recent studies show that precise control of this coupling enables unprecedented manipulation of droplet arrays^33,45^ and phase-change processes,^46^ opening up novel opportunities in other disciplines, such as materials processing and pharmaceutical applications.

### Droplet Oscillation

The oscillatory behavior of acoustically levitated droplets results from the complex interplay among surface tension, acoustic radiation force, and viscous damping and exhibits rich nonlinear dynamics that can significantly influence thermal and mass-exchange characteristics. The fundamental droplet oscillation frequency follows a modified Rayleigh–Lamb relationship^41,47,48^ of

2 $$f_{0}={\left(8\sigma /\pi^{2}\rho_{l}D_{0}^{3}\right)}^{1/2}\left[1+0.32We_{a}^{0.7}-0.11Oh^{1.3}\right]$$

where *We*_*a*_ is the acoustic Weber number (ρ_*g*_ *U*_*a*_^2^ *D*_0_/σ), *Oh* (= μ_*l*_/(ρ_*l*_ *σ D*_0_)^1/2^) is the Ohnesorge number, and the subscripts 0, *l*, *g*, and *a* represent initial, liquid, gas, and acoustic, respectively. High-speed imaging reveals three distinct oscillation regimes based on the Δ*D*/*D*_0_ ratio:^48–50^ (i) Δ*D*/*D*_0_ < 0.05: surface-tension dominance at small amplitude, axisymmetric vibrations at sound pressure levels (*SPL*) less than 145 dB, (ii) 0.05 < Δ*D*/*D*_0_ < 0.15: mode coupling and nonlinear shape deformations featuring harmonic generation, and (iii) Δ*D*/*D*_0_ > 0.15: chaotic oscillations preceding droplet breakup. The oscillation amplitude shows a conclusive resonance near *f* ≈ 0.7*f*_0_ at which the acoustic radiation force and droplet response reach a π/2 phase shift, coinciding with the maximum energy transfer.^41,48^ The sound pressure level in dB is determined from 20*l*og10·(*A*_0_c) + 74, with higher *SPL* enhancing both droplet deformation and evaporation. The oscillation behavior is further influenced by the ambient temperature, leading to an increase in oscillation damping ratio by 25–40% for a temperature variation between 20 and 80 *°*C due to reduced surface tension and altered viscosity.^51–53^

The droplet oscillation dynamics is modified by acoustic streaming through two competing mechanisms: (i) enhanced damping from viscous dissipation in the Stokes layer (δ ≈ (2ν/ω)^1/2^) and (ii) energy input from periodic vortex shedding at the droplet equator.^54–56^ The oscillation behavior of binary mixtures indicates a notable dependence on composition. For instance, the ethanol–water droplets exhibit oscillation amplitudes 30–50% larger than those of pure component droplets, which can be attributed to mode coupling induced by Marangoni stress.^13,57^ The oscillation spectrum typically exhibits several peaks corresponding to spherical-harmonic modes from 2 to 6. The second mode is predominant at low SPL owing to its minimal energy requirement and strong coupling with acoustic radiation pressure.^19^ The dominant mode denotes the lowest-order axisymmetric oscillation mode (spherical-harmonics index).

In Figure 7, the droplet oscillation results were obtained using a single-axis acoustic levitator operated at 19 kHz at atmospheric ambient conditions for fluids with varying vapor pressure.^14^ High-speed images presented in Figure 7 depict that the dominant oscillations enhance internal mixing by up to 45% when compared to stationary droplets while simultaneously modifying the evaporation rate through surface-area modulation.^14^ However, the higher modes become more prominent as SPL exceeds 150 dB.^40,48,49^ The flow field induced by oscillation also influences the thermal boundary layer around the droplet, resulting in periodic thickness variations from 15 to 25% that can have a considerable impact on heat-transfer rates.^48,52^

**Figure 7:**
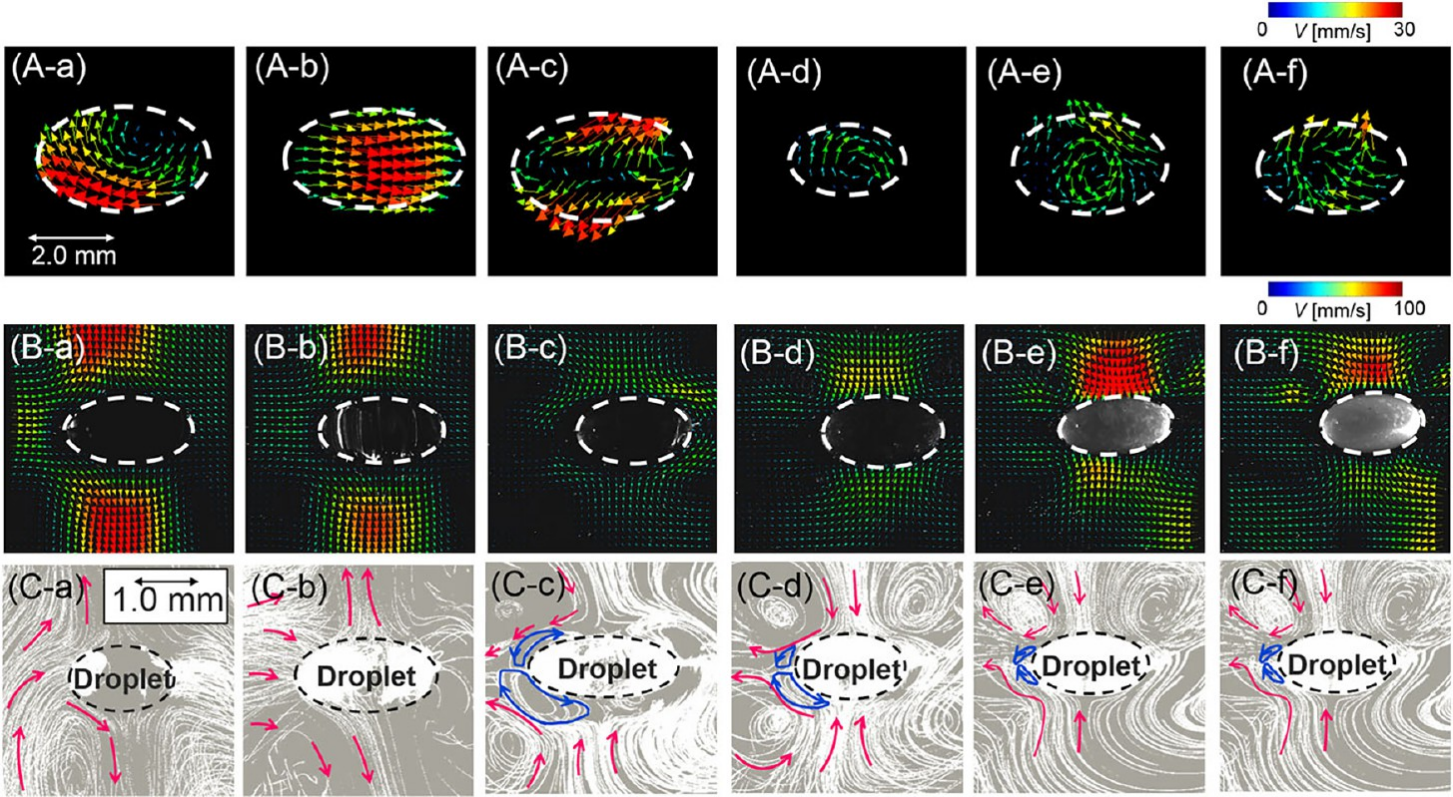
Internal and external flow structure and dynamics of acoustically levitated volatile droplets. (A) Flow field inside the droplet, (B) flow field outside the droplet, and (C) flow dynamics around the droplet showing vortices. Volatile droplets: (a) decane, (b) nonane, (c) octane, (d) heptane, (e) hexane, and (f) pentane. Reproduced with permission from ref [Bibr ref14], 2025, AIP Publishing.

Oscillatory effects are particularly evident during the final stages of droplet evaporation as the rising solute concentration induces nonlinear stiffening of the droplet surface. It is accompanied by a 15–30% increase in oscillation frequency.^41,48^ The acoustic field, through acoustic-wave-droplet coupling and droplet oscillation mechanism, significantly influences transport phenomena (flow patterns, thermal energy exchange, and mass exchange) both inside and outside the droplets. Figure 8 demonstrates the relationship between the modulation frequency (*f*_mod_) and the oscillation frequency (*f*_*n*_) for the fourth–seventh mode oscillations. The oscillation frequency is directly proportional to half of the modulation frequency. These trends were shown by considering a 50 wt % glycerin aqueous solution levitated in a phased-array acoustic levitator setup operated at 40 kHz, atmospheric ambient conditions, and by using laser-induced fluorescence and particle image velocimetry techniques.

**Figure 8:**
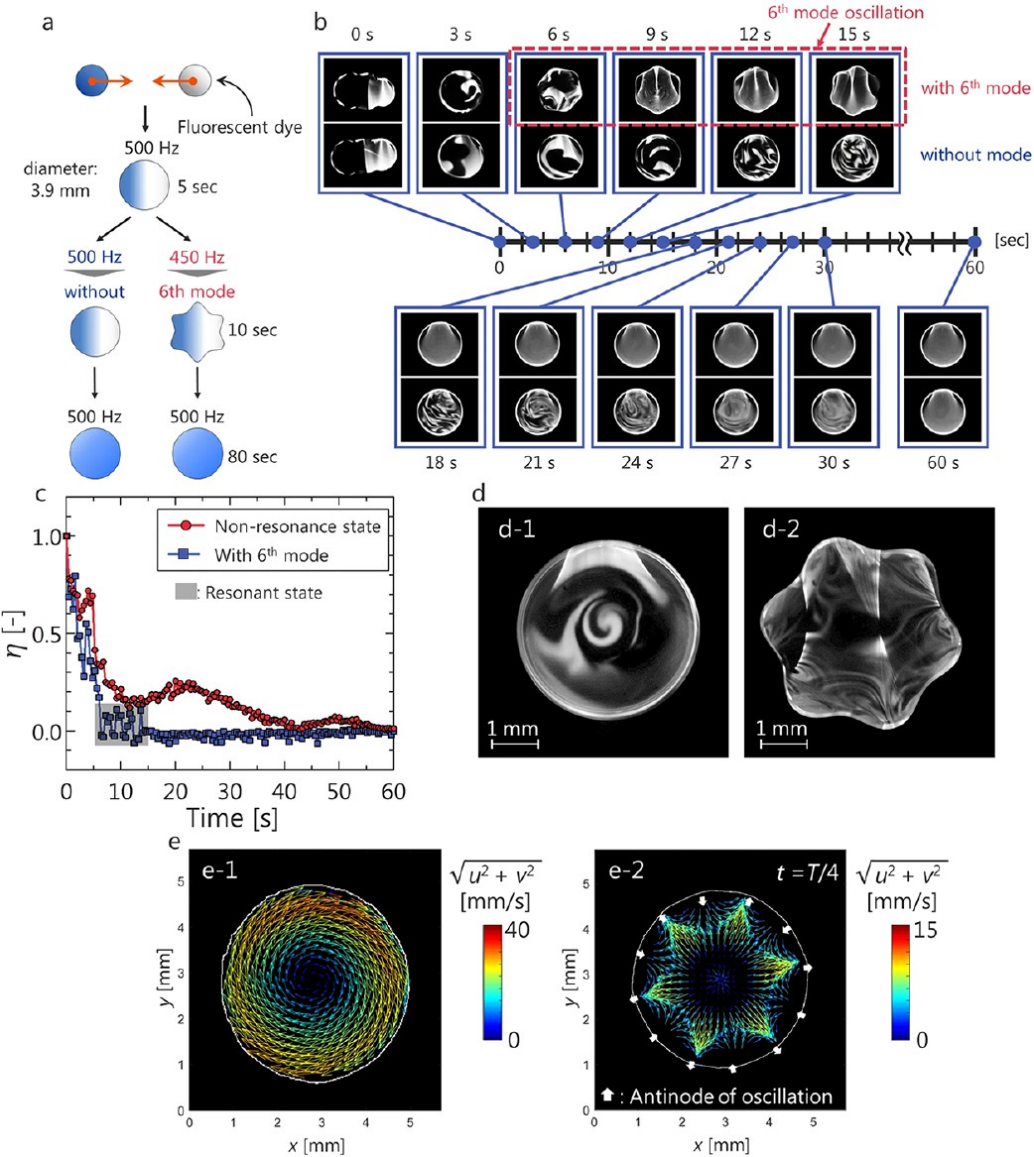
Relation between modulation frequency and oscillation frequency of an acoustically levitated droplet, (a) experimental scheme, (b) comparison of mixing behavior, (c) transition of mixing parameter, (d) mixing patterns at different oscillation modes, and (e) flow structure comparison. Reproduced from ref 35. Available under a CC-BY 4.0 license. Copyright 2018, Ayumu Watanabe, Koji Hasegawa, and Yutaka Abe.

### Acoustic Levitation: Droplet-Discrete-Phase Interplay

Colloidal droplets find applications in various fields, including pharmaceuticals, food processing, spray coating, and energy.^58,59^ Here, the colloidal droplets refer to a mixture of a pure fluid and discrete-phase particles, typically nanometer- or micrometer-sized, at different concentrations. Thus far, liquid-in-liquid mixture droplets, both miscible and immiscible, have been extensively investigated.^60–62^ Unlike single-phase droplets, the dispersion of discrete-phase (solid) particles could alter the internal flow structure and, in turn, the thermal and mass exchange between the droplet and the surroundings. Therefore, it is important to understand not only the flow- and evaporation dynamics of a droplet dispersed with nanoparticles but also to gain insights on the final drop morphology after the evaporation of solvents.^58,63^ Nonetheless, studies on acoustically levitated solid-in-liquid mixture droplets are very limited in the literature. In the case of discrete-phase dispersed droplets, particle sedimentation at the solid surface is a major challenge. Therefore, the acoustic levitation method is considered the best alternative for suspending droplets containing nanoparticles, thereby eliminating the solid–liquid interface and enabling investigation of their drop morphology and evaporation.

The evaporation dynamics of an acoustically levitated droplet can be broadly classified into two stages: in the first stage, the diameter of the droplet decreases due to evaporation of solvents, and in the second stage, the slowing down of the evaporation rate due to the accumulation of discrete particles, which leads to crust formation on the surface of the acoustically levitated droplet. The classical *d* ^2^-law is followed only during the first stage, where droplet shrinkage takes place. Therefore, during the initial stage of evaporation, the dynamics of the colloidal droplet can be correlated with that of a pure droplet, as it also follows the *d* ^2^-law as that of the pure droplet.^51^ After that, the discrete particles precipitate on the droplet’s outer surface and lead to the crust formation and a decrease in the evaporation rate.^64^ The shape of the crust formation depends on the particle concentration. At high particle concentrations, internal circulation about the levitated axis is driven by acoustic streaming and ultimately forms a ring-like structure. However, at a lower concentration, evaporation dominates, and a bowl-like structure is formed.^65^ Density stratification induced by acoustic streaming causes particle migration due to the force imbalance between the continuous phase and the discrete phase, and thus, a bowl shape is created.^66–68^ Therefore, the presence of nanoscale/microscale particles significantly influences the evaporation dynamics and the structure of acoustically levitated droplets.

## Acoustic Field Influence on Transport Phenomena

Droplet dynamics are inherently linked to the local flow field surrounding the droplet and to thermal and mass exchange between the droplet and the surrounding medium. Typically, these interactions occur in practical combustors used for power generation. Similar scenarios are likely to exist in other scientific applications as well. Therefore, it is essential to understand the transport phenomena governing the droplet dynamics in an acoustic field.

### Acoustic Streaming

Acoustic streaming plays a vital role in droplet evaporation, colloidal droplet morphology, and the circulation of suspended nanoparticles within droplets. Acoustic streaming is also one of the important parameters for droplet levitation over a surface, which is known as surface acoustic-wave-driven streaming. Surface acoustic waves (SAW) have been widely used for droplet migration and trapping in microchannels.^69^ Surface acoustic-wave-driven streaming generates a streaming-induced drag force, which, together with hydrodynamic forces and van der Waals attractive forces, collectively governs droplet coalescence, trapping, and transport.^70^ Acoustic streaming plays a crucial role in particle aggregation by generating a hydrodynamic pressure gradient force induced by SAW excitation.^71^ Furthermore, SAW can also lead to the formation of Cassie–Wenzel wetting transition on a superhydrophobic surface through liquid penetration into nanogrooves.^72^ Like acoustic levitation, SAW can provide contactless manipulation via acoustic streaming, with the advantage of rewritability of the droplet floating on an immiscible fluid layer.^73^ Acoustic streaming can be converted into acoustic streaming vortices by varying the transducer voltage, thereby enabling precise control of droplets on a rewritable fluid path.^74^ Acoustic black holes (specially engineered structures to trap and concentrate acoustic-wave energy from the progressive waves) have high-energy regions that produce acoustic streaming and the acoustic radiation force, which can be used for the manipulation of droplets.^75^ Therefore, it is important to develop an in-depth understanding of acoustic streaming and the factors that influence it. The steady flow of fluid and/or particles within the droplet (see Figures 7(a) and 8(e)), caused by the absorption or reflection of acoustic energy, is known as acoustic streaming.^76^ The acoustic streaming can be broadly classified into three types: (1) Ekart, (2) Schlichting, and (3) Rayleigh streaming. Ekart streaming arises from attenuation of acoustic amplitude.^77^ Schlichting streaming occurs within the viscous boundary layer when a solid wall is present and is driven by the shear force.^78^ The Rayleigh streaming arises in the bulk of fluid outside the viscous boundary layer due to net momentum transfer.^79^ The last two types of streaming are used for the micromanipulation of nanoparticles via acoustic streaming^80^ and for self-assembly.^76^ Acoustic streaming, in turn, can significantly influence thermal and mass exchange between the droplet and its surroundings. For instance, the toroidal vortices (see Figure 9) induced around the droplet by the acoustic field can disturb the fuel vapor boundary layer.^81^

**Figure 9:**
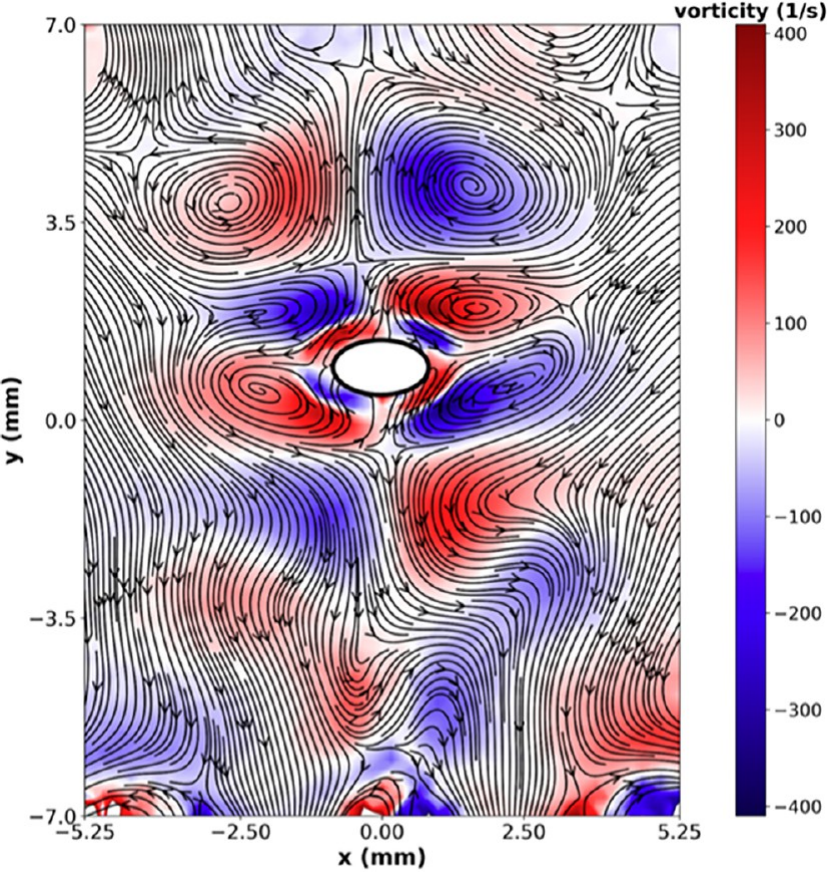
Acoustic streaming around a levitated 2 mm diameter methanol droplet in ambient air. Freely available for download from ref 81.

Acoustic streaming plays an important role by increasing the convective transport around the levitated drop, which ultimately increases the mass transfer, which is often characterized by a higher Sherwood number (*Sh*).^51,55^ Such an effect is important for avoiding local vapor saturation and for augmenting evaporation rates, especially for high-volatility fuels.^32,44^ In the context of combustors, high-frequency acoustic fields (20–100 kHz) can enable rapid mixing of fuel and air within 500 *μs* for droplet diameters less than 100 μm, which directly influences the combustion efficiency.^82^ These results underscore the need for a comprehensive analysis of acoustic streaming in the *AL* method for fuel droplet evaporation, given its direct bearing on combustion performance in aerospace, automotive, and energy applications.

Early seminal work by Nyborg^17^ and Whymark^18^ established the fundamentals of acoustic streaming near the droplet boundaries and paved the way for in-depth theoretical and experimental investigations. The equilibrium shape and dynamics of acoustically levitated droplets have been extensively investigated to determine droplet stability and the flow around them. Trinh and Hsu^19^ reported an early perspective on the equilibrium shapes of droplets in acoustic fields. The dynamic pressure component of *F*_rad_ is called Bernoulli pressure and arises from the velocity field of the standing wave. It contributes to the asymmetric deformation of levitated droplets (i.e., flattening of droplet spherical shape into oblate spheroids).^30^ Acoustic streaming in levitated droplets generates complex three-dimensional flow structures and profoundly influences heat and mass transfer processes. The streaming velocity field emerges from nonlinear interactions between the acoustic-wave and droplet interface, following the generalized formulation: *u*_s_ = (3/16)­(*U*_0_^2^/*c*)­[1 – (*r*/*R*)^2^ + *A*(∂*T*/∂*r*)], where *A* includes both thermal and viscous effects.^52,54,83^ This causes two types of circulation: (i) internal toroidal flows with speeds of 0.5–2.5 mm/s assisting in internal mixing and (ii) external vortices that reach 1.5–2 droplet diameters into the gas phase around them.^14,57,84^ The internal flow observations were made using silver-based colloidal water droplets under standard atmospheric conditions in a single-axis acoustic levitator operated at 19 kHz with a 2.5 mm droplet diameter.^54^ The external flow-field structures were obtained under similar conditions using water mist as a tracer and the particle image velocimetry technique. Similarly, Yarin et al.^41,51^ demonstrated that acoustic streaming enhances mass and heat-transfer rates in single- and binary-component droplets and compared with classical diffusion-driven models.

Under acoustic levitation, the Stokes boundary-layer thickness expressed as δ = (2ν/ω)^1/2^ typically ranges between 10 and 50 μm for levitation frequencies varying between 20 and 40 *kHz* and directly affects the transport (mass and heat) coefficients through the following relationships:^85^ *Sh* ≈ 2 + 0.6*R*e^1/2^ *Sc* ^1/3^ and *Nu* ≈ 2 + 0.6*R*e^1/2^ *Pr* ^1/3^. The boundary-layer dynamics greatly improve heat and mass transfers, as characterized by the increase in the Nusselt number (*Nu*) by up to 4.5-fold^14,52^ and the Sherwood number (*Sh*) by a factor of about 4.^55,86^ These observations were derived from studying the dynamics of volatile colloidal droplets such as decane, nonane, etc., with nylon particles in a single-axis acoustic levitator operated at 19 kHz under ambient atmospheric conditions. Under similar conditions, the acoustic streaming phenomenon generates distinct flow patterns, with inner streaming velocities reaching 0.5–2 mm/s due to toroidal circulation within the droplet.^14,51^ In contrast, outer streaming creates counter-rotating vortices with characteristic dimensions of λ/4,^54,87^ as seen in Figure 9. In this context, the acoustic attenuation effect governs the dissipation of ultrasound energy in the medium. While it indirectly influences evaporation by modulating acoustic streaming forces, it does not directly represent fluid mixing or thermal exchange processes.^33,43^ Furthermore, the thermal coupling creates three characteristic zones: a cold core region with temperature depression up to 2.5 °C due to evaporative cooling,^53,88^ a hot boundary layer with thermal gradients exceeding 10^3^ K/m,^14,89^ and a vapor enrichment zone extending 0.5–1.5 droplet diameters with relative humidity > 90%.^55,86^ The resulting mass flux follows a relationship of *ṁ*″ = *h*_*m*_·(*c*_s_ – *c*_∞_)·[1 + 0.12·(SPL – 140)^1.3^], where the sound pressure level modification accounts for acoustic enhancement effects.^41,55^ Pressure variations up to 150 Pa generated by the acoustic field further modify the droplet evaporation characteristics through its influence on local vapor saturation conditions.^85,86^

Recent studies reveal that acoustic streaming alone accounts for about 60–80% of the total heat-transfer enhancement in levitated droplets dominated by the convective transport.^14,89^ The vortex circulation strength peaks at intermediate frequencies (*f* = 30 kHz) due to optimal boundary-layer coupling,^33,85,89^ while temperature dependencies cause about 15–25% velocity variations in nonvolatile droplets (water) when compared to volatile (hydrocarbon) droplets.^51,52,90^ In binary mixtures, compositional effects are especially strong because Marangoni stresses from surface-tension gradients cause flow reversals at random times, changing the movement pattern.^13,54,57^ High-speed tomographic PIV images show that the instantaneous acoustic streaming field exhibits substantial harmonic content, altering the boundary-layer thickness and enhancing turbulent transport.^12,54,90^ The droplet deformation (*AR* > 1.3) produces anisotropic streaming that preferentially enhances transport along the major axis while creating stagnation zones near the poles.^12,14,56^ Further, recent studies have identified coherent structures in the streaming field that elucidate the 40–60% transport enhancement observed in pulsed acoustic fields with the help of dynamic mode decomposition,^45,84,87^ while numerical simulations demonstrate the essential role of three-dimensional effects in precisely predicting heat and mass transfer coefficients.^88^

### Acoustic Streaming Influence on Particle Migration

As highlighted in the previous section, the circulation pattern inside an acoustically levitated colloidal droplet is significantly governed by acoustic streaming, which arises from the interaction of the high-frequency acoustic field with the surrounding gas.^51^ The droplet shape oscillations further strengthen and modulate the internal flow patterns.^91^ These circulation currents play a crucial role in transporting particles within the droplets. As reported in the literature,^66^ acoustic-streaming-driven particle migration leads to the development of density stratification, producing a force imbalance that weakens the internal recirculation, slows the evaporation rate, and eventually promotes the formation of a bowl-shaped droplet morphology. Since acoustic streaming is a direct consequence of the finite-amplitude acoustic pressure field,^45,92^ the evaporative flux becomes nonuniform along the droplet surface: evaporation is enhanced at the equator and suppressed near the poles. This uneven evaporation, combined with gravity, drives particle migration from the equatorial region toward the poles.^67^ In addition, the swirling flow generated by acoustic streaming induces rotational motion of particles around the levitator axis, and the resulting centrifugal redistribution contributes to the formation of ring-shaped particle deposits.^65^ The dispersion of discrete particles alters the fluid viscosity, and the fluid properties, in turn, influence the droplet dynamics. For instance, the viscous boundary-layer dynamics introduces additional complexity, with the attenuation coefficient exhibiting a strong correlation with frequency and fluid properties.^24,33^

### Acoustic Field—Thermal and Mass Transport

As highlighted earlier, the acoustic levitation method has been particularly beneficial for investigating droplet evaporation nonintrusively. Zaitone^93^ demonstrated that evaporation rates for deformed droplets adhere to a modified *d* ^2^-law, with significantly accelerated mass loss relative to near-spherical droplets. This increased evaporation rate arises from the greater surface-area-to-volume ratio and intensified acoustic boundary-layer effects near the droplet equator, where the convective heat-transfer coefficient attains local maxima. The inferences were derived based on the pure water droplets in a single-axis acoustic levitator operated at 58 *kHz* under atmospheric ambient conditions. Recent studies^14,54,94^ have further revealed the intricate internal and external flow transitions in evaporating droplets, elucidating the coupling between acoustic waves and thermocapillary effects and their impact on droplet stability and evaporation. Furthermore, in evaporating droplets, the evaporation rates are governed by *SPL* primarily through two mechanisms, namely, (a) Bernoulli pressure-induced surface stretching increases vapor diffusion coefficients by up to 4 · *D*_0_,^37^ and (b) acoustic streaming starting above 150 dB enhances convective mass transfer.^32^ Experimental data showed that the evaporation rate enhancement can vary from 300 to 500% when the *SPL* is increased from 140 to 160 dB.^26^ The thermophysical mechanism of levitated droplets encompasses complex behavior governed by a thermodynamic energy equation, focusing on energy balance and coupled mechanisms

3 $$\rho_{l}c_{p,l}V_{d}\left(\frac{dT_{d}}{dt}\right)=hA_{s}\left(T_{g}-T_{d}\right)-L_{v}\left(\frac{dV_{d}}{dt}\right)+Q_{a}c$$

where *Q*_ac_ = α_*a*_ *cp*_0_^2^ *V*_*d*_/(ρ_*g*_ *c*_*g*_) represents the thermal contribution of acoustic heating.^29,31^ The infrared thermography measurements highlight that temperature depressions between ambient gas and droplet (Δ*T* = *T*_g_ – *T*_*d*_) can range from 2.5 °C for water to 4.2 °C for ethanol, with characteristic relaxation times varying between 3 and 15 s depending on the droplet composition.^29,33^ These thermal gradients arise from competing heat-transfer mechanisms between evaporative cooling and convective heating and, in acoustically levitated systems, from acoustic energy deposition.^38^ The observed temperature differences and relaxation dynamics are intrinsically linked to interfacial transport phenomena, particularly within the viscous boundary layer that develops at the droplet-ambient gas interface.^95^ This boundary layer plays a crucial role in governing momentum and energy transfer, as it directly influences both the convective heat-transfer coefficient and the evaporation rate.^96^ In the *AL* method, the boundary layer assumes additional importance due to its interaction with the oscillatory acoustic field.^97,98^ The attenuation of acoustic energy within this boundary layer contributes to the overall energy balance and may affect both the droplet’s thermal equilibrium and its evaporation kinetics.^99^ These results highlight that boundary-layer dynamics are essential for accurately modeling levitated droplet systems, particularly when focusing on temperature depressions and relaxation time scales.^42^

Rednikov and Riley^100^ demonstrated that when a droplet is subjected to an ultrasonic standing wave, the resulting steady streaming flow dramatically alters heat-transfer dynamics. Under nonreactive conditions, acoustic microstreaming creates a toroidal vortex adjacent to the droplet, as experimentally detected by Trinh and Robey.^101^ When the droplet is heated, however, buoyancy forces couple with this streaming to create new vortex structures. For instance, a heated cylinder in an acoustic field develops dual counter-rotating vortex pairs: one above the cylinder due to arrested acoustic jets and one below due to thermal buoyancy opposing the downward jet.^100^ This restructuring of the flow increases thermal mixing and decreases the temperature gradients at the interface. The convective heat transfer, characterized by Nusselt number, initially decreases with the rising buoyancy parameter (as flow expands and surface gradients are reduced); however, later it increases monotonically as buoyancy-dominated vortices enhance scalar transport.^100^ In liquid droplets, internal circulation driven by viscosity contrasts and displacement from pressure nodes further reshapes the streaming pattern, wrapping vortices in “doughnut-shape” around the droplet. This amplifies surface heat fluxes by up to 300% when compared to stagnant environments.^100,101^ The streaming Reynolds Number (*Re*_s_ = *U*_*s*_^2^/*Ω* ν) is a dimensionless parameter that characterizes the relative strength of inertial forces to viscous forces within the acoustic flow field. At high acoustic amplitudes, when *Re*_s_ ∼ *O*(10^2^), the acoustic-streaming-induced turbulence dominates over buoyancy, optimizing evaporation rates in fuel droplets.^47^

Argyri et al.^102^ greatly extended the capabilities of acoustic levitation by coupling it with noncontact magnetic resonance imaging and spectroscopy, enabling molecular-level investigations of dynamic evaporation and chemical reactions within levitated droplets. In addition to experimental methods, computational tools are also offering greater mechanistic insight into acoustic streaming, heat and mass transfer, and droplet behavior under levitation conditions. Numerical investigations by Bänsch and Götz,^88^ Doß and Bänsch,^103^ and Wang and Hardalupas^81^ have successfully modeled the complex transient processes of droplet evaporation in acoustic levitation, droplet lifetime, shape deformation, and the internal flow behavior accurately. Such computational models are highly useful for gaining additional insights into experimental data and for predicting system responses under different acoustic and thermal conditions.

## Droplet Breakup

The utility of acoustic levitation in combustion studies has been greatly exemplified by investigations into the ignition, combustion, and microexplosion behavior of multiphase droplets. Brotton et al.^104^ examined the ignition behavior of acoustically levitated jet fuel (JP-10)-based colloidal droplets dispersed with aluminum nanoparticles in a single-axis acoustic levitator at about 125 kPa and ignited using a CO_2_ laser. Significant modifications to the fuel ignition delay and burning intensity were observed. Likewise, Zhang et al.^105^ reported that acoustic excitation influences *n*-heptane droplet combustion and offered key insights into the coupling between acoustic-induced droplet oscillations and combustion kinetics. The role of the acoustic field in evaporation, along with thermal and mass exchange, is discussed in the previous section. Here, the role of the acoustic field in droplet breakup and recent advancements are discussed.

The breakup of fuel droplets is an integral step toward achieving complete combustion and, in turn, higher combustion efficiency.^106,107^ Droplet breakup increases heat transfer and mixing due to the high surface-area-to-volume ratio.^108^ The presence of discrete particles can further affect the droplet breakup process.^106,109,110^ From a fuel standpoint, the main purpose of adding metal particles to liquid fuels is to increase the energy density, thermal conductivity, and, in turn, the combustion efficiency while reducing the pollutant emissions.^111^ A higher degree of droplet breakup was obtained due to boiling induced by nanoparticle aggregation-based nucleation sites, leading to microexplosion phenomena.^112,113^ The increased intensity of droplet breakup is partly also due to enhanced heat absorption by the nanoparticles.^112^ Particle size is also an important factor affecting the droplet breakup. Nonetheless, investigations into the effects of nanoparticles on the breakup of acoustically levitated droplets have been limited in the literature. Therefore, relevant studies on the breakup dynamics of acoustically levitated droplets, particularly under thermal and acoustic excitations, are briefly discussed.

Experimental investigations by Basu et al.,^30^ Wei et al.,^114^ and Aoki and Hasegawa^115^ elucidated the threshold conditions for droplet breakup, acoustic pressure, and thermal disturbances that trigger disruptive events. Hasegawa and Abe^116^ investigated transport phenomena in acoustically levitated droplets and identified distinctive acoustic streaming patterns (highlighted in previous sections) that influence droplet breakup and coalescence. The breakup dynamics of acoustically levitated droplets are complex, governed by the balance between surface tension and acoustic radiation pressure. When the acoustic Weber number (*We*_ac_ = ρ_*g*_ *U*_ac_^2^ *D*_0_/σ) exceeds the critical value (typically in the range of 0.5–1.2 depending on the liquid characteristics), the droplet breaks up catastrophically through a sequence of stages established in the literature.^30,117,118^ High-speed imaging demonstrates that droplet breakup begins with the formation of axisymmetric surface waves (*n* = 2 mode) at the equator of the droplet, with wavelength λ_w_ ≈ (2πσ/ρ_*l*_ω^2^)^1/3^.^30,119^ These surface instabilities grow exponentially with time constant, leading to the formation of well-defined lobe structures that detach as secondary droplets.^117,118^ The child droplet-size distribution is log-normally distributed to the function: *d*_50_ ≈ 0.15·*D*_0_·*W*e_ac_^–0.4^, where the mean prefactor shows large liquid viscosity dependence through the Ohnesorge number and *D*_0_^1/2^.^118–120^

Figure 10 illustrates the top-view observation of a water droplet breakup at different time instances. The droplet initially deformed and spread radially (*t* = −0.28 ms). Subsequently, the capillary wave was initiated from the edge of the droplet (*t* = −0.14 ms), and the droplet breakup started (*t* = 0 ms). The capillary wave propagated from the edge to the center of the droplet. Finally, the child droplets were dispersed radially through capillary waves, and the droplet was completely disintegrated into child droplets (at *t* = 5.00 ms).^115^ Sophisticated numerical simulations using level-set methods have also reported similar fine-scale topological evolution during droplet breakup. The numerical results also indicate that acoustic pressure distribution induces stress maxima in localized regions at the poles of the droplets that drive capillary wave propagation.^117,121^ In another study by Saroj and Thaokar,^122^ the effect of droplet diameter on droplet breakup is examined through the acoustic Weber number. A critical acoustic Weber number (*We*_ac_ ≈ 1.36) for the fuel droplet was investigated beyond which the droplet breakup phenomena starts.

**Figure 10:**
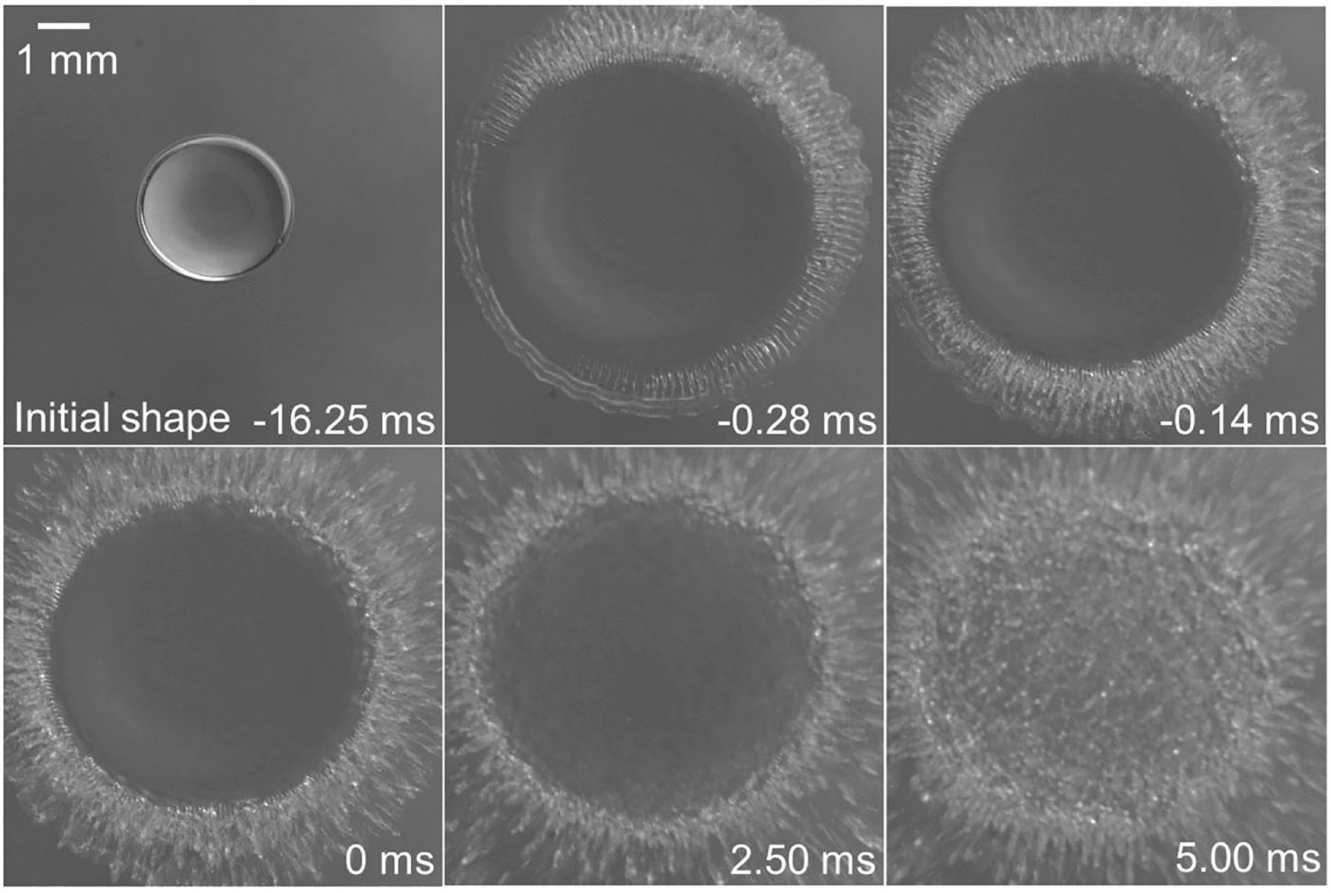
Breakup of the water droplet at different time instances (initial conditions: *d* = 3.5 mm and Δ*P =* 0.57 kPa). Reproduced from ref 115. Available under a CC-BY 4.0 license. Copyright 2020, K. Aoki and K. Hasegawa.

Figure 11 depicts pure ethanol droplets with high vapor pressure (5.3 kPa at 25 °C) showing simple evaporation without any secondary breakup for all droplet sizes (droplet sizes: 300–500 μm), regardless of the addition of ceria nanoparticles (up to 0.5 wt %). In stark contrast, pure *n*-dodecane droplets having low vapor pressure (17.65 Pa at 25 °C) illustrate two different breakup modes: (1) Kelvin–Helmholtz (KH) instability-driven pinching for initial droplets below a critical size (*d*_0_ < 450 μm), where shear forces from acoustic streaming eject child droplets from the equator and (2) catastrophic breakup (CB) for larger initial droplets (*d*_0_ > 450 μm), characterized by acoustic flattening, formation of a thin central membrane, and rim disintegration due to a modified Weber number exceeding stability thresholds (driven by a 28% reduction in surface tension at elevated temperatures).^45^ The latest developments in ultrasonic levitator design enable precise control over droplet breakup thresholds via dynamic frequency modulation, thereby enabling selective production of monodisperse droplet ensembles with lower geometric standard deviations for applications requiring precise size control.^29,33,123^

**Figure 11:**
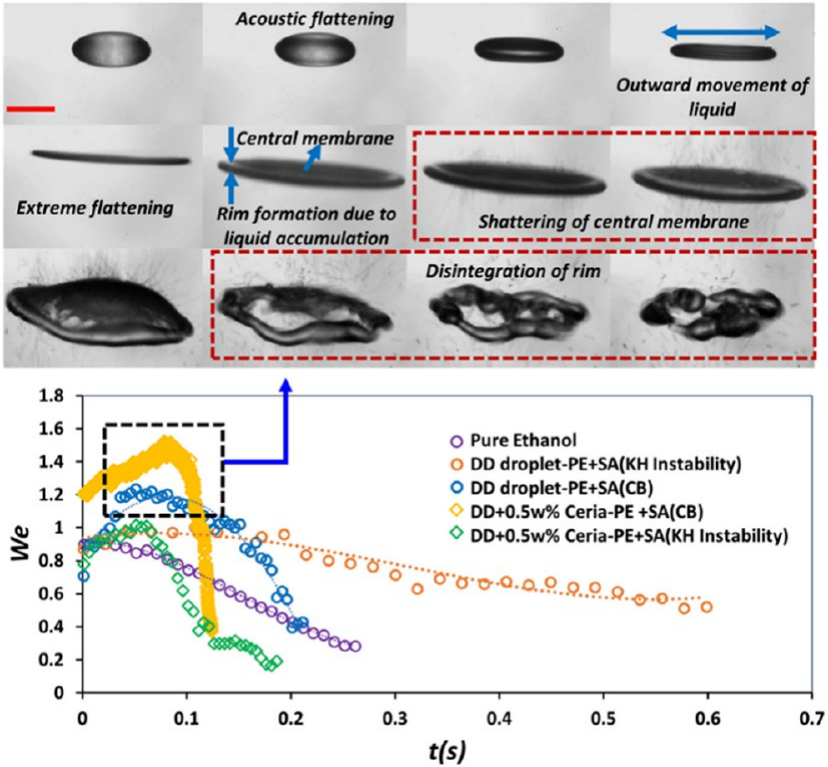
Influence of Weber number on the droplet breakup dynamics. *Top*: Visual representation of mechanisms involved in the droplet breakup dynamics. *Bottom*: Time variation of droplet dynamics as a function of Weber number for pure ethanol (PE), pure dodecane (DD), and nanoparticle-laden (NP) dodecane droplets that exhibit secondary atomization (*SA*). Reproduced with permission from ref [Bibr ref34], 2025, AIP Publishing.

## Droplet Coalescence

In acoustically levitated droplets, coalescence (i.e., merging of multiple smaller droplets to form a larger droplet) is governed by the balance of acoustic radiation force, surface-tension force, and the viscous dissipation force. When two droplets approach each other, the air film between them is displaced quickly, as explained by the Reynolds lubrication equation, ∂*h*/∂*t* = (*h* ^3^/12 μ)∇^2^ *p*, where *h* is the film thickness and μ represents the liquid dynamic viscosity.^119,121^ Typically, the air film displacement occurs in three stages, namely, (1) initial slow displacement (*t*_1_ ≈ 10–50 ms) where the film thins from approximately 10 μm to 100 nm, (2) rapid displacement (*t*_2_ ≈ 1–5 ms) during which the intermolecular (van der Waals) forces become dominant, and (3) finally, breakup of the air film (*t*_3_ < 1 ms) leading to the bridge formation between the liquid droplets.^124,125^ The acoustic field significantly modifies this process through several mechanisms, first, the acoustic radiation pressure creates a localized stress concentration that can accelerate the air film displacement by up to 30%, second, the acoustic streaming generates vortical flows (ω ≈ 5–200 s^–1^) that enhance momentum transfer, and third, the standing-wave structure imposes a preferential orientation on the merging droplets.^37,116,123^ As highlighted in previous sections, vortical flows are induced by the acoustic streaming inside and outside the colloidal droplets.^54^

Honda et al.^126^ studied the effect of fluid properties on the coalescence of binary droplets (e.g., pure ethanol, glycerol-water, and pure glycerol). They demonstrated that controlled reduction of acoustic focal points induces horizontal opposing flows in ethanol and glycerol, with ethanol exhibiting slightly higher velocities, which are driven by surface deformation (refer to Figure 12). The flow reversals are similar to oscillation-induced patterns reported earlier. More importantly, the droplet viscosity governed the oscillation damping in low-viscosity fluid (ethanol, ν = 1.387 × 10^6^ m^2^/s), sustaining long-lived oscillations. However, high-viscosity fluids (pure glycerol, ν = 720.0 × 10^6^ m^2^/s) exhibit minimal deformation due to viscous dampening, reviving Lamb’s theory. In binary mixtures, composition gradients generate Marangoni stresses (τ_M_ ≈ 0.01 – 0.05 N/m) induced by the composition gradient, accelerating or decelerating coalescence depending on component volatility.^37,116,123^ Honda et al.^126^ reported three factors that dominate the mixing kinetics: (1) initial contact area expansion during coalescence (evident in glycerol droplets), (2) oscillation intensity higher in low-viscosity droplets, and (3) oscillation duration (ethanol > glycerol-water > glycerol).

**Figure 12:**
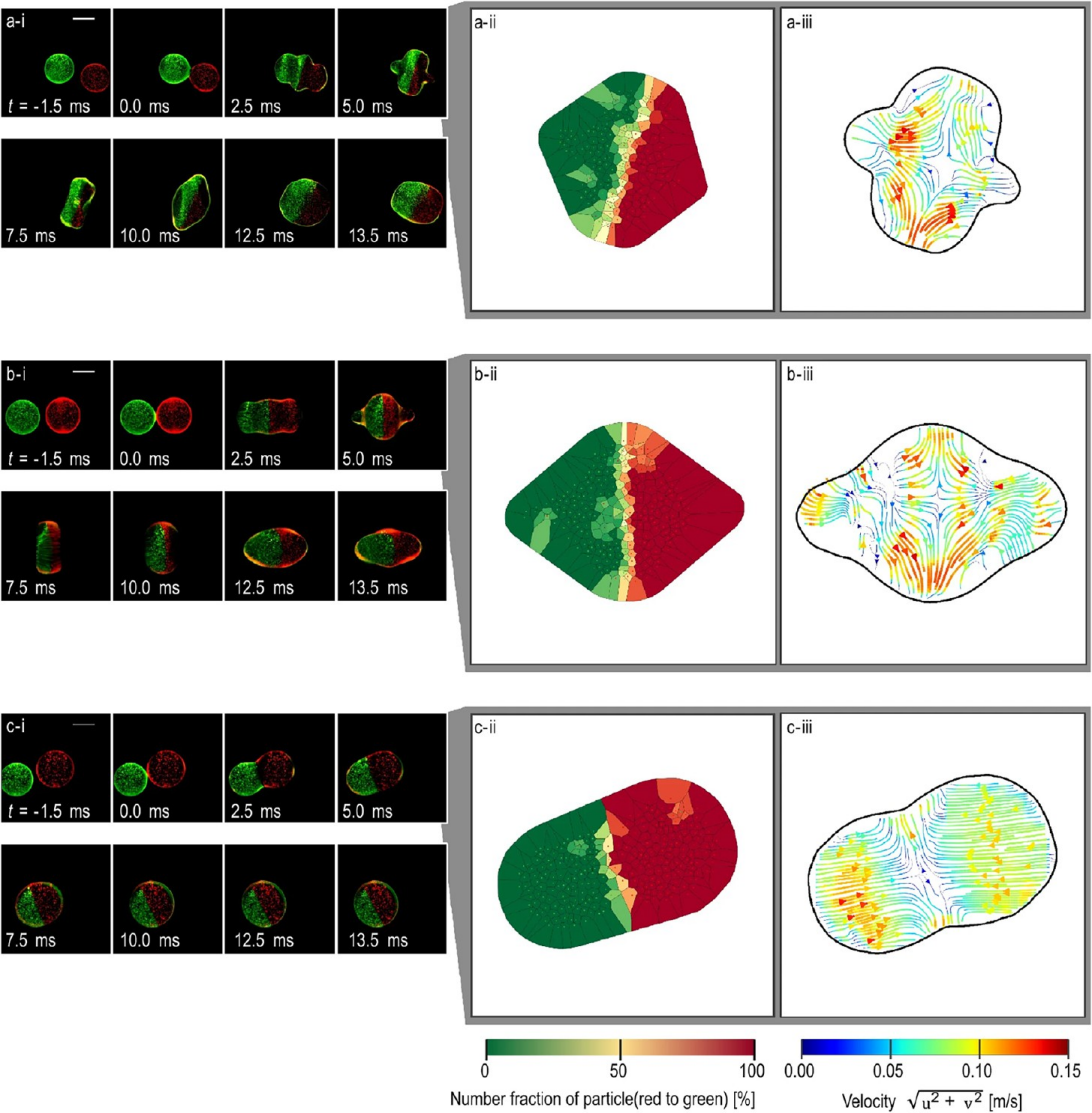
Effect of fluid properties on the coalescence of binary droplets. (a) Ethanol droplets, (b) 33 wt. % glycerol-water solution droplets, and (c) pure glycerol droplets. Photographs, the number fraction of particles, and flow-field details are shown for each case. Reproduced from ref 126. Available under a CC-BY 4.0 license. Copyright 2023, Kota Honda, Kota Fujiwara, Koji Hasegawa, Akiko Kaneko, and Yutaka Abe.

In addition to fluid properties, the droplet size affects coalescence. For instance, in water droplets of initial diameters of 1 mm, the liquid bridge forms with an initial radius ≈ 0.1 D and the bridge growth is influenced by surface tension and density.^124,125^ The droplet coalescence dynamics exhibit a strong dependence on droplet-size ratio, where unequal-sized droplets (*D*_1_/*D*_2_ > 1.5) amalgamate 20–30% faster than equal-sized pairs owing to enhanced curvature-driven flows.^116,124,127^ The process is also affected by the droplet temperature, which in turn, influences the viscosity (Δμ ≈ −2% per °C for water) and alteration of surface tension (Δσ ≈ −0.15 mN/m per °C). A further increase of temperature by 20 °C reduces the total coalescence duration by about 35–45%.^119,123^ These results highlight that fluid properties significantly influence droplet coalescence, which is substantially altered when discrete-phase particles are dispersed within the droplet.

## Discussion Summary

Fundamental studies of acoustic field and liquid droplet interactions using an acoustic levitator are presented and discussed. In this context, significant advancements and new insights into droplet dynamics, acoustic field-droplet interactions, thermal and mass-transport characteristics, droplet breakup dynamics, and droplet coalescence mechanisms are discussed. The acoustic levitation method uniquely employs noncontact suspension of isolated liquid droplets for fundamental analysis under controlled experimental conditions. For this reason, acoustic levitation has been increasingly employed to investigate droplet evaporation processes in the absence of heterogeneous nucleation sites. The discussion highlights that droplet studies using the acoustic levitation method require attention to three major aspects: (i) the influence of acoustic streaming, (ii) the droplet breakup threshold, and (iii) the conditions that enable droplet coalescence.

First, the critical aspect involved in acoustic levitation, i.e., dominance of acoustic streaming and oscillation dynamics over droplet behavior, is discussed with recent advancements. Acoustic streaming markedly enhances thermal and mass-exchange rates by thinning the boundary layers around droplets, thereby promoting rapid heat and mass transfer with the surrounding medium, which is highly beneficial in practical applications.^128–130^ Streaming-induced toroidal vortices change droplet shape, evaporation rate, and internal mixing, and are highly sensitive to acoustic parameters such as frequency, amplitude, and ambient pressure.^131^ Despite extensive perusal, precise control and measurement of streaming effects across diverse fluids, especially multicomponent and colloidal droplets, remain challenging.^34,132^ Acoustic levitation, in particular, alters the droplet’s thermal equilibrium by inducing energy deposition and evaporative cooling, thereby directly influencing droplet lifespan and stability. So far, the complex interactions among acoustic intensity, frequency, and droplet composition remain challenging, especially in multicomponent systems, underscoring the need for further analysis to precisely measure and optimize these effects.^133^ The coupling between acoustic fields and thermal and mass-transport mechanisms has markedly improved droplet evaporation rates, often exceeding predictions from traditional diffusion-driven models.

Second, the droplet oscillation dynamics in acoustic fields exhibit complex behaviors, especially near-resonant conditions, with significant nonlinearities that influence thermal and mass-exchange processes. The nonlinear relationship among oscillation amplitudes, sound pressure level, and droplet size provides a basis for forecasting droplet breakup conditions.^21,47^ However, further research into these nonlinear phenomena, specifically at higher acoustic intensities and across various droplet configurations, is necessary to fully leverage the capabilities of complete acoustic levitation in practical engineering applications.

Third, critical thresholds for droplet breakup, which depend on the acoustic Weber number, fluid viscosity, and temperature, provide valuable insights into droplet dynamics, which is crucial for practical combustors.^134^ The established relationship between acoustic conditions and droplet breakup provides a practical indication for specific atomization control in fields such as pharmaceuticals, fuel processing, and advanced material synthesis. However, the complications posed by acoustic-driven instabilities necessitate further experimental and numerical studies, particularly those involving multiphase and volatile fluids.^103^ Experimental studies^114,115^ have demonstrated that the breakup phenomena (for example, Kelvin–Helmholtz instability) arise from rapid pressure fluctuations at droplet interfaces under intense acoustic fields. Similarly, new insights into droplet coalescence mechanisms have emerged through the noninvasive acoustic levitation method, albeit with a few limitations.

Despite these improvements, vital limitations and challenges remain. The transient behaviors of multicomponent mixtures, nanofluids, and emulsions under acoustic levitation conditions remain incompletely understood.^135,136^ These research gaps can be addressed through coordinated experimental-computational approaches and improve the predictive capabilities and practical applicability of acoustic levitation in emerging fields.

## Limitations and Future Research Directions

In earlier sections, the effectiveness of acoustic levitation as a scientific tool for studying droplet characteristics, the interplay among the transport mechanisms involved, factors that strongly affect droplet dynamics, and insights gained through acoustic levitation into the dynamics of discrete particle-dispersed droplets are discussed in detail. Nonetheless, studies on acoustically levitated solid-in-liquid mixture droplets are still very limited and further experimental and computational studies are warranted. Despite considerable advances, several challenges remain in acoustically levitated droplet studies, and further research in these areas is warranted to deepen existing knowledge. Some of them are listed below:

- Multicomponent and multiphase droplet behavior: Under the acoustic field, fully resolving the transient evaporation and phase-change dynamics of complex multicomponent, multiphase (colloidal) droplets that recently gained significant attention and are commonly explored in energy applications is essential. Specifically, phase separation and microscale heterogeneity remain incompletely understood,^135–138^ and addressing them would significantly broaden the applicability of acoustic levitation to emerging scientific fields.
- Acoustic Streaming and Coupling Effects: It is challenging to accomplish explicit control and quantification of acoustic streaming flows and their coupling with droplet dynamics. The nonlinear coupling between the acoustic field and droplet, especially for multicomponent/multiphase droplets, makes it difficult to predict or design thermal and mass transport.^115,130,132^ The complex interplay between acoustic frequency and amplitude and droplet volatility and composition remains poorly characterized, and more experimental evidence is required to develop a theoretical framework to maximize the benefits.^131,133^ The majority of the inferences reported are still specific to the acoustic levitation system considered, the type of fluids (volatile, nonvolatile), and are limited to atmospheric ambient conditions. Further studies at ambient conditions, relevant to practical applications, would be beneficial.
- Droplet Size and Multidroplet Limitations: The conventional single-axis axisymmetric acoustic levitator can only suspend relatively small droplets due to stability limits, with a maximum droplet size of a few millimeters in normal gravity.^39^ Recent advances in transducer array technology and acoustic phase shaping have begun to break through this envelope, enabling the levitation of larger droplets, or even multiple droplets. However, these systems still remain in the development phase and are not yet widely used. Further advancements in this direction will broaden the scope of studies of acoustically levitated droplets.
- Nonlinear Oscillations and Droplet Breakup Thresholds: The dynamics of the oscillation and fragmentation of droplets at high acoustic amplitudes have not been fully understood yet. Closer to resonance conditions, levitated droplets exhibit complex nonlinear oscillations and instabilities, and quantitative thresholds for droplet fragmentation (i.e., breakup) as a function of acoustic parameters and fluid properties are yet to be well-established.^114,115,139^ More in-depth research is warranted to gain a deeper understanding and control over these acoustic parameters, particularly under varying fluid viscosities, surface tensions, and ambient conditions.
- Coalescence and mixing dynamics: In the case of multidroplet levitation, the process of coalescence and subsequent mixing is only partially understood. It is experimentally challenging to capture the rapid interface dynamics and internal mixing during the merging of two droplets in midair. Recent developments in high-speed imaging and selective visualization have provided some insights into coalescence behavior in acoustic fields.^116,126^ However, overall knowledge of the interaction between acoustic forces and mixing or phase separation during coalescence remains incomplete. This deficiency hinders the optimum use of acoustic levitation in understanding droplet–droplet interactions and reactive droplet amalgamation.

## Conclusions

Acoustic levitation, as a scientific tool, offers significant advantages for studying the thermal and mass-exchange characteristics of liquid droplets suspended in an acoustic field, which are directly relevant to practical engineering applications. In this review, the fundamental aspects of the balance of forces that enable stable suspension, competing axial and radial forces on droplets, and interactions between the acoustic field and multiphase droplets (i.e., colloidal and emulsion droplets) are presented and discussed. Followed by three critical aspects involved in acoustic levitation, acoustic streaming influence, droplet breakup threshold, and droplet coalescence are discussed in detail. Based on the discussion presented, the following key points warrant further research.

- Acoustic levitation offers itself as an excellent scientific tool for understanding the droplet evaporation characteristics in a nonintrusive way. However, the current design of the widely used single-axis, axisymmetric acoustic levitator limits the droplet size, position, and droplet count. Further advances are needed to address the challenges posed by the multiphase droplets.
- Acoustic streaming significantly enhances thermal and mass exchange between the droplet and its surroundings by reducing boundary layers and promoting convective mixing, which, in turn, directly influences the evaporation rates. However, controlling streaming flows via acoustic field parameters has been challenging. In addition, the dispersion of particles in droplets, for example, nanoparticles, is further complicated by the nonlinear droplet-acoustic field coupling. Therefore, the thermal and mass transport of single- and multiphase droplets differ considerably.
- Droplet oscillation dynamics in acoustic fields exhibit nonlinear resonance effects, which are of central importance to predict droplet stability, droplet breakup thresholds, and thermal/mass interactions with the surroundings. Precise control of these oscillation dynamics in multiphase droplets is central to understanding the final droplet morphology given the droplet’s broad application potential.
- Acoustic levitation enables a deeper understanding of droplet breakup, revealing significant thresholds and fragmentation mechanisms as functions of the acoustic Weber number, fluid viscosity, and thermal conditions.
- Recent advances have integrated particle velocimetry techniques, infrared thermography, and magnetic resonance imaging with acoustic levitation, with great success, to expand our understanding of internal flows of droplets dispersed with particles/bubbles, thermal distribution, phase-change dynamics, and drying mechanisms. Experimental investigations are supplemented by numerical simulations to gain a more mechanistic understanding of transient droplet dynamics in acoustic fields. Continued development of robust computational models remains important for improving the predictive accuracy.

In summary, acoustic levitation is an extremely versatile and valuable scientific tool, offering promising avenues for advancing fundamental understanding and practical skills in droplet dynamics, especially when progressing from single-phase droplets to multiphase droplets. Nevertheless, significant knowledge gaps persist, particularly regarding transient effects in complex fluid systems, such as multicomponent mixtures, emulsions, and particle-laden droplets. Addressing these issues through interdisciplinary research will broaden technical expertise, deepen insights, and widen the scientific applications of acoustic levitation. Further research is warranted to address existing limitations, expand the operating boundaries, integrate with other advanced diagnostic tools, and build a comprehensive experimental database to refine the numerical models.
